# Brucellosis in Southeast Asia: A Systematic Review of Epidemiological Evidence, Surveillance Gaps, and cgMLST‐Based Insights Into Genetic Relatedness

**DOI:** 10.1155/tbed/9792290

**Published:** 2026-10-06

**Authors:** Qiuju Yang, Xiaoan Cao, Linghui Yuan, Na Zhang, Zhenjun Li, Zhiguo Liu

**Affiliations:** ^1^ Department of Zoonosis Prevention and Control Yunnan Provincial Key Laboratory for Natural Focal Disease Control and Prevention Yunnan Institute of Endemic Disease Control and Prevention Kunming 650500 China; ^2^ Veterinary Clinical Service Center State Key Laboratory of Animal Disease Control and Prevention Lanzhou Veterinary Research Institute Chinese Academy of Agricultural Sciences Lanzhou 730046 China caas.cn; ^3^ Branch of Biosafety National Key Laboratory of Intelligent Tracking and Forecasting for Infectious Diseases National Institute for Communicable Disease Control and Prevention Chinese Center for Disease Control and Prevention and Chinese Academy of Preventive Medicine Beijing 102206 China chinacdc.cn

**Keywords:** brucellosis, cgMLST, Southeast Asia, surveillance, systematic review

## Abstract

Brucellosis is an important zoonosis affecting public health and livestock production in Southeast Asia, but its regional epidemiology remains incompletely characterized. In this study, PubMed, Embase, Web of Science, and Scopus were searched for primary studies from the 11 Southeast Asian countries that reported serological findings, molecular detection, bacterial isolation, or *Brucella* species identification in human or animal populations. Publicly available PubMLST records were also analyzed using the 1764‐locus *Brucella* core‐genome multilocus sequence typing scheme. Across the eligible studies, reported crude seropositivity proportions ranged from 0.72% to 5.40% in humans and from 0.24% to 8.83% in animals. Molecular evidence included PCR positivity rates of 3.36% in goat vaginal swabs from Thailand and 39.2% in a selected mixed‐livestock population from Indonesia, together with the identification of *Brucella melitensis*, *B. abortus*, and *B. suis* in several Southeast Asian countries. These findings provide epidemiological evidence of brucellosis in several countries. cgMLST analysis of the 24 available profiles from Malaysia and Thailand revealed close genetic relatedness among several isolates. Malaysian and Thai *B. melitensis* isolates differed by fewer than 11 alleles within the main cluster and by 41–81 alleles relative to isolates from India and the United Kingdom. Two Thai *B. abortus* isolates differed at only one locus and by 68–150 alleles from isolates from Bangladesh, India, and Bolivia, whereas five Malaysian *B. suis* isolates differed by 7–78 alleles, indicating limited genetic diversity within the Malaysian cluster. Additional genomic and epidemiological data are needed to determine whether genetically similar isolates are epidemiologically linked. Brucellosis appears endemic in parts of Southeast Asia, but important gaps in published data remain. Integrated One Health surveillance is needed to improve regional risk assessment and control.

## 1. Introduction

Brucellosis is a globally distributed zoonosis caused by facultative intracellular bacteria of the genus *Brucella*. It is more common in low‐ and middle‐income countries, particularly where extensive livestock production is widespread [1]. A recent evidence‐based analysis estimated that ~2.1 million human cases occur worldwide each year [2]. Human infection commonly presents with nonspecific symptoms, including fever, sweating, fatigue, and malaise, which may delay clinical recognition and diagnosis [3]. Transmission occurs primarily through direct contact with infected animals or contaminated reproductive materials and through the consumption of unpasteurized dairy products [4]. Consequently, livestock farmers, slaughterhouse workers, veterinarians, laboratory personnel, and consumers of unpasteurized milk are considered high‐risk populations in endemic areas [4].

Brucellosis is recognized among the top seven neglected zoonoses [5]. Its nonspecific clinical presentation and potential to cause chronic disease contribute to delayed diagnosis, underreporting, and sustained health and socioeconomic consequences [6]. Human and animal infections have been documented in several Southeast Asian countries [7], but the available evidence differs considerably with respect to geographic coverage, host species, diagnostic capacity, and study period. Regional epidemiological patterns are influenced by livestock production systems, animal movement, ecological conditions, and access to medical and veterinary services [8]. A recent investigation in Myanmar documented ongoing bovine infection and limited farmer awareness [9]. Molecular surveillance in northeastern Thailand also identified infection in goat herds [10]. A regional systematic review was therefore undertaken to summarize the available epidemiological and microbiological evidence, identify methodological and geographic gaps, and place the available Southeast Asian cgMLST profiles in a global context. The review focused on the scope and limitations of the available evidence and on the level of inference supported by the genomic data.

## 2. Materials and Methods

This review covered the 11 sovereign states of Southeast Asia: Brunei Darussalam, Cambodia, Indonesia, the Lao People’s Democratic Republic (Lao PDR), Malaysia, Myanmar, the Philippines, Singapore, Thailand, Timor‐Leste, and Vietnam. Eligible studies were primary epidemiological or microbiological investigations reporting serological findings, molecular detection, bacterial isolation, or Brucella species identification. PubMed, Embase, Web of Science, and Scopus were searched from inception through May 1, 2026. The review was conducted and reported in accordance with PRISMA 2020, and the study‐selection process is summarized in Figure S1. The structured evidence synthesis focused on empirical studies; one review article and one case report were retained for contextual completeness and were not included in the Mixed Methods Appraisal Tool (MMAT) appraisal.

The search strategy combined terms related to brucellosis, epidemiological or diagnostic evidence, and the 11 Southeast Asian countries as follows: (*Brucella*  ^∗^ OR brucellosis) AND (seroprevalence OR serology OR antibody OR PCR OR molecular OR isolation OR culture) AND (Brunei OR Cambodia OR Indonesia OR Laos OR “Lao PDR” OR Malaysia OR Myanmar OR Philippines OR Singapore OR Thailand OR “Timor‐Leste” OR “East Timor” OR Vietnam). The search strategy was adapted to the specific requirements of each database. In addition, the reference lists of eligible articles and relevant regional reviews were screened to identify further primary studies.

### 2.1. Study Selection and Data Extraction

Duplicate records were removed using EndNote X9 and checked manually. Two reviewers (Linghui Yuan and Na Zhang) independently screened the titles and abstracts and assessed full texts against the eligibility criteria; disagreements were resolved by consensus or consultation with a third reviewer (Qiuju Yang). Data were extracted using a standardized form covering study characteristics, population or exposure, animal species and production system, sample size, diagnostic methods, test results, and reported *Brucella* species or biovars. Distinct subgroups were recorded separately when they were independently interpretable.

### 2.2. Data Synthesis and Methodological Quality Assessment

Diagnostic methods were grouped as serological tests (RBT, SAT, and ELISA), molecular tests (BCSP31‐PCR, IS711‐PCR, and qPCR), or bacterial isolation. Study‐level numerators and denominators were retained, and crude positivity rates were calculated when available. Subgroup analyses by country, host species, and diagnostic method were considered, but the small number of comparable studies and substantial heterogeneity in study populations, sampling, diagnostic approaches, outcome definitions, and study periods precluded meaningful statistical pooling. Findings were therefore summarized descriptively without statistical weighting.

Methodological quality was assessed using the MMAT, Version 2018. Prevalence, surveillance, and descriptive case, clinical, or isolate studies were assessed as quantitative descriptive studies, whereas cohort and analytical cross‐sectional or retrospective studies examining exposure–outcome associations were assessed as quantitative nonrandomized studies. The five design‐specific criteria were rated as “Yes,” “No,” or “Can’t tell.” No overall numerical score was calculated, and the appraisal was used to inform interpretation of the evidence.

### 2.3. cgMLST Data Retrieval and Comparative Analysis

cgMLST data for isolates originating from 11 Southeast Asian countries were retrieved from the PubMLST database (https://pubmlst.org/). Eligible isolates with complete and interpretable records were included in subsequent analyses, together with available information on the species or strain type, cgMLST allelic profiles, host, and other relevant metadata. Twenty‐four records met these criteria: two *B. abortus* profiles from cattle in Thailand; 16 *Brucella melitensis* profiles from Malaysia (*n* = 13) and Thailand (*n* = 3); and one unclassified *Brucella* sp. profile from Thailand and five *B. suis* profiles from humans in Malaysia. No eligible profiles were identified for the remaining nine Southeast Asian countries. Allelic comparisons were based on the 1764‐locus *Brucella* cgMLST scheme [11]. Selected profiles from outside Southeast Asia were included solely to provide a comparative allelic context. Minimum spanning trees were generated using GrapeTree 2.0 [12]. The analysis was used to assess the genetic relatedness among the available Southeast Asian *Brucella* isolates.

## 3. Results

### 3.1. Study Selection and Methodological Quality of the Included Studies

The initial search identified 252 records, of which 124 were duplicates. Screening of the remaining 128 records excluded 108 studies, leaving 20 eligible studies. A supplementary targeted search identified 14 additional studies, resulting in 34 publications being retained in the review (Figure S1). Methodological quality was assessed for the 32 empirical studies using MMAT 2018; one review article and one case report were not assessed because MMAT was not applicable to these publication types. The empirical evidence comprised 16 quantitative descriptive studies and 16 quantitative nonrandomized studies. Limitations most frequently involved sampling and population representativeness. Outcome and diagnostic measurements were appropriate across the assessed studies, whereas five nonrandomized studies did not adequately address potential confounding. Study‐level assessments are provided in Supporting Information, Table S1.

### 3.2. Human Serological Findings

Human serological findings were available for Malaysia, Thailand, Indonesia, and Vietnam (Figure 1 and Table 1). Reported crude seropositivity proportions were 5.40% (87/1612) in Malaysia, 2.01% (3/149) in Indonesia, 1.72% (7/406) in Vietnam, and 0.72% (3209/444,427) in Thailand. In the Vietnamese study, 64 of 406 samples were reactive by RBT, whereas seven showed weak reactivity in the subsequent IgM/IgG flow assay; Table 1 therefore presents 7/406 and specifies this diagnostic limitation. No eligible human serological study was identified for Brunei Darussalam, Cambodia, Lao PDR, Myanmar, the Philippines, Singapore, or Timor‐Leste. These estimates were not directly comparable because of differences in study populations, sampling criteria, study periods, and diagnostic algorithms.

**Figure 1:**
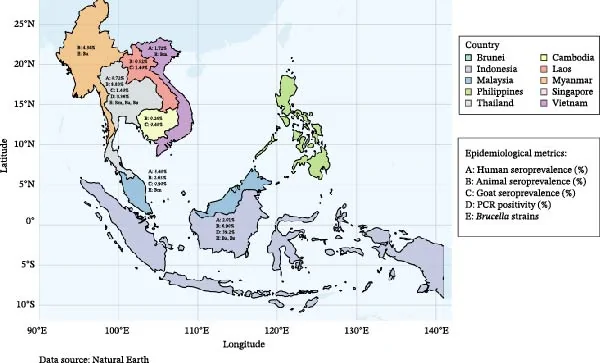
Geographic distribution of epidemiological evidence for brucellosis in Southeast Asia. The map summarizes evidence identified in the review: (A) human serological findings; (B) animal serological findings; (C) goat serological findings; (D) study‐specific PCR findings; and (E) *Brucella* species reported in eligible studies (Ba, *B. abortus*; Bm, *B. melitensis*; and Bs, *B. suis*). Countries without eligible evidence should be represented as areas with no data rather than zero prevalence. Base‐map data were obtained from Natural Earth; the displayed extent is 90°E–140°E and 10°S–25°N.

**Table 1:** Descriptive epidemiological evidence for human and animal brucellosis in Southeast Asia.

Country	Human serological positivity	Animal serological positivity	Goat serological positivity	PCR positivity rate	Reported *Brucella* species
Brunei	ND	ND	ND	ND	ND
Cambodia	ND	4/1681 = 0.24%	0.40%	ND	ND
Indonesia	3/149 = 2.01%	95/1377 = 6.90%	ND	40/102 = 39.2%	*B. abortus*; *B. suis*
Lao PDR	ND	36/6924 = 0.52%	1.40%	ND	ND
Malaysia	87/1612 = 5.40%	11,101/419,645 = 2.65%	0.90%	ND	*B. melitensis*
Myanmar	ND	5/115 = 4.34%	ND	ND	*B. abortus*
Philippines	ND	ND	ND	ND	ND
Singapore	ND	ND	ND	ND	ND
Thailand	3209/444,427 = 0.72%	1015/11,501 = 8.83%	1.40%	11/327 = 3.36%	*B. melitensis*; *B. abortus*; *B. suis*
Timor‐Leste	ND	ND	ND	ND	ND
Vietnam	7/406 = 1.72%^a^	ND	ND	ND	*B. melitensis*

*Note*: ND, no eligible data identified in the searched sources. Serological proportions are crude descriptive values derived from the available reports and are not pooled national prevalence estimates. Human and animal values are not directly comparable. PCR results are study‐specific and were obtained from goat vaginal swabs in Thailand and blood samples from selected goats and sheep in Indonesia.

^a^In Vietnam, 64/406 samples were RBT reactive, but 7/406 showed weak reactivity in the subsequent IgM/IgG flow assay. The species column summarizes eligible epidemiological and microbiological studies and does not include the separate PubMLST analysis.

### 3.3. Animal Serological Findings

Animal serological data were available from Cambodia, Indonesia, Lao PDR, Malaysia, Myanmar, and Thailand (Figure 1 and Table 1). Crude seropositivity ranged from 0.24% in Cambodia (4/1681) to 8.83% in Thailand (1015/11,501), with estimates of 6.90% in Indonesia, 4.34% in Myanmar, 2.65% in Malaysia, and 0.52% in Lao PDR. Differences in animal species, production systems, sampling, study periods, and diagnostic methods limited direct comparisons. No eligible data were identified for the remaining five countries. In addition, goat‐specific seropositivity was 0.40% in Cambodia, 0.90% in Malaysia, and 1.40% in both Lao PDR and Thailand.

### 3.4. Study‐Specific PCR Findings

Animal PCR findings were reported from Thailand and Indonesia. *Brucella* DNA was detected in 11 of 327 goat vaginal swabs in Thailand (3.36%) and in 40 of 102 blood samples from goats and sheep in Indonesia (39.2%). These findings were derived from selected animal populations and should not be interpreted as population‐level prevalence estimates. No eligible animal PCR data were identified for other Southeast Asian countries.

### 3.5. Reported *Brucella* Species

Based on the available studies, the only *Brucella* species identified in Malaysia and Vietnam was *B. melitensis*; *B. melitensis*, *B. abortus*, and *B. suis* were reported in Thailand; *B. abortus* was reported in Indonesia and Myanmar; and *B. suis* was isolated from pigs in Indonesia (Table 1). No eligible report provided species‐level identification for Brunei Darussalam, Cambodia, Lao PDR, the Philippines, Singapore, or Timor‐Leste. These results summarize the available published evidence and do not indicate the absence of unreported species. The separate PubMLST analysis also included Malaysian *B. suis* profiles; these database records are not included as epidemiological studies in Table 1.

### 3.6. Availability and Composition of cgMLST Profiles

PubMLST contained 24 Southeast Asian records with usable cgMLST profiles, all originating from Malaysia or Thailand (Table 2). The dataset comprised two Thai *B. abortus* cattle profiles (cgST 366 and 741); 16 *B. melitensis* profiles from humans (*n* = 10) or goats (*n* = 6), including three from Thailand and 13 from Malaysia; and one unclassified Thai *Brucella* sp. profile (cgST 390) and five Malaysian human *B. suis* profiles (cgST 1–5). This geographic concentration reflects limited database coverage and sequencing availability and should not be taken as representative of the genomic diversity across Southeast Asia.

**Table 2:** Characteristics of the 24 Southeast Asian *Brucella* records included in the PubMLST cgMLST analysis.

*Brucella* species	Country	Number of records	Number of cgST	Host (number)	Year	Reported cgST labels
*B. abortus*	Thailand	2	2	Cattle	2006	366, 741
*B. melitensis*	Thailand	3	2	Human (*n* = 3)	2006	376;1476, 1476
	Malaysia	13	8	Human (*n* = 7); goat (*n* = 6)	2009–2015	544;1154;1155, 545, 546, 547, 1138, 1139, 1154;1155, 1155
*Brucella* sp.	Thailand	1	1	Unknown	1963	390
*B. suis*	Malaysia	5	5	Human	2009–2015	1, 2, 3, 4, 5

### 3.7. Phylogenetic Placement of Southeast Asian *Brucella* Isolates in a Global Context

The global cgMLST analysis placed *Brucella* isolates from Malaysia and Thailand into four groups (I–IV). Group I contained all *B. melitensis* isolates, Group II contained the two *B. abortus* isolates from Thailand, Group III contained five *B. suis* isolates from Malaysia, and Group IV contained a single isolate from Thailand. The regional isolates showed varying degrees of genetic relatedness to isolates from Bangladesh, India, Bolivia, the United Kingdom, Spain, and Costa Rica (Figure 2). Selected closely related isolates were then examined in a minimum spanning tree to compare within‐species allelic differences.

**Figure 2:**
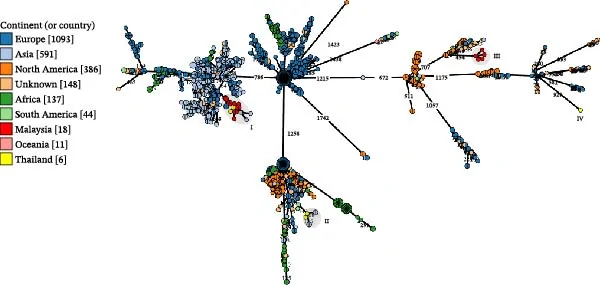
cgMLST‐based allelic relationships among Southeast Asian and selected global *Brucella* profiles. Colors indicate geographic origin, and bracketed values indicate the number of profiles. Roman numerals I–IV denote the four species‐associated groups represented by the available Southeast Asian profiles. Branch labels indicate the number of differing cgMLST loci. Profiles from outside Southeast Asia were included solely as selected comparators; the figure does not establish transmission direction, geographic origin, or importation.

### 3.8. cgMLST Allelic Differences Within Southeast Asian Groups and Relative to Selected Global Comparator Profiles

The *B. melitensis* isolates from Malaysia (*n* = 13) and Thailand (*n* = 3) largely clustered together (Figure 3). Within the main cluster, adjacent profiles in the minimum spanning tree differed by fewer than 11 cgMLST loci. The Thai isolate ST 1476 differed from ST 376/1476 by 11 alleles. Among the Malaysian isolates, ST 545 and ST 546 differed from ST 376/1476 by four and three alleles, respectively, whereas ST 547 differed from the Malaysian node ST 544/1154/1155 by four alleles. In contrast, ST 1138 and ST 1139 differed from ST 376/1476 by 127 and 78 alleles, respectively. The cluster also showed genetic relatedness to the UK isolate ST 727 and the Indian isolate ST 670, differing by 41 and 81 alleles, respectively. The two Thai *B. abortus* isolates differed at only one cgMLST locus. Their closest included isolates from Bangladesh, India, and Bolivia differed by 68, 115, and 150 alleles, respectively (Figure 3). The five Malaysian *B. suis* isolates differed from one another by 7–78 alleles and were separated from the Costa Rican isolates by 454 alleles. A Thai *Brucella* isolate assigned to cgMLST type 390 differed from the United Kingdom isolate by 292 alleles.

**Figure 3:**
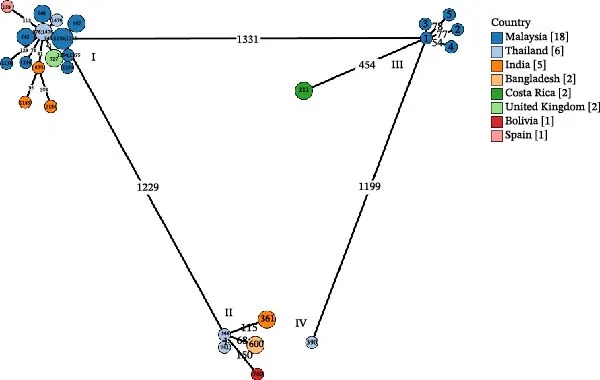
Minimum spanning tree of cgMLST allelic differences among Southeast Asian and selected global comparator profiles. Colors indicate geographic origin, and bracketed values indicate profile counts. Roman numerals I–IV denote the four species‐associated groups. Branch labels indicate the number of differing cgMLST loci. Short allelic distances indicate genomic relatedness within the cgMLST scheme and should not be interpreted as evidence of direct transmission, source attribution, or transmission direction.

## 4. Discussion

This review summarizes serological, microbiological, and molecular evidence on brucellosis in Southeast Asia, including findings from human and animal serology, bacterial isolation and species identification, PCR detection, and cgMLST analysis. The findings document infection at the human–animal interface, but the available evidence remains insufficient to define the broader One Health epidemiology of brucellosis in the region. The purpose was not to estimate pooled regional prevalence but to describe the scope and limitations of the available evidence across countries, hosts, diagnostic approaches, and genomic datasets.

Reported human seropositivity proportions ranged from 0.72% to 5.40%, but these values were derived from different study populations and cannot be compared with national estimates. In Vietnam, 64 of 406 samples were RBT‐reactive, whereas seven showed weak reactivity in the follow‐up flow assay; the absence of seroconversion further constrained interpretation [13]. In Malaysia, 10 of 184 serum samples from clinically suspected patients were antibody positive, and most positive patients reported occupational contact with animals [14]. These findings show that case selection and diagnostic algorithms influence the reported positivity. In settings with limited disease awareness, restricted laboratory access, and inadequate follow‐up, low reported positivity may also reflect underdiagnosis. Differences among serological assays also affect their interpretation. RBT, SAT, ELISA, and sequential testing algorithms differ in diagnostic performance, cutoff definitions, and intended use; therefore, some between‐study variation may reflect testing strategy rather than true epidemiological variation. Most reports did not provide the information needed to adjust study‐level estimates for test sensitivity and specificity, so no such adjustment was made.

Occupational exposure is an established determinant of human brucellosis [15], and direct contact with animals and handling of aborted materials are recognized risk factors [16]. However, the magnitude of the occupational risk could not be quantified because many studies did not report specific work activities, informal employment, sex, or household responsibilities. Thailand had the lowest crude human seropositivity proportion, whereas national small‐ruminant surveillance documented marked herd‐level seropositivity in selected years and locations [17]. This finding does not imply high human prevalence but supports targeted surveillance among populations with frequent small‐ruminant contact. In Lao PDR, goat seropositivity varied according to the production system, farm characteristics, and geographic location [18]. A survey of goats in Cambodia also identified geographic variation among the sampled provinces [19]. Future studies should collect detailed exposure information for farmers, slaughterhouse workers, veterinarians, dairy workers, informal traders, and individuals involved in animal parturition.

Molecular detection provides direct evidence of *Brucella* DNA, but its yield depends strongly on the specimen type and sampling design. In Indonesia, *B. abortus* DNA was detected in 40 of 102 selected goats and sheep in a mixed‐livestock setting [20]. The reported positivity reflects the sampled population and cannot be extrapolated to the provincial or national levels. This result supports sampling multiple host species in mixed farming systems.

The available species‐level evidence included *B. melitensis*, *B. abortus*, and *B. suis*, although their regional distributions remain incompletely defined. Human isolates from Malaysia have included *B. melitensis* [21], and collections from Thailand have included *B. melitensis*, *B. abortus*, and *B. suis* from human and animal hosts [22]. *B. suis* has also been isolated from pigs in Indonesia [23]. Future surveillance should include livestock, buffaloes, wildlife‐livestock interfaces, and mixed farming systems. Climatic and environmental factors may alter risk through animal movement, reproductive material management, and informal trade, although these pathways were not directly assessed in the included studies [24].

Genotyping studies in Asia have assigned many *B. melitensis* isolates to the East Mediterranean lineage and have identified structured diversity within *B. abortus* [25]. Malaysian MLVA data similarly assigned most human *B. melitensis* isolates to the East Mediterranean lineage [26], whereas Vietnamese isolates showed VNTR relatedness to strains from several geographic regions [27]. In the present cgMLST analysis, several Malaysian and Thai isolates showed relatively small allelic differences from selected isolates from India, the United Kingdom, and other countries. This finding is consistent with previous genomic studies that placed Malaysian *B. melitensis* isolates within broader Asian lineages [28, 29]. However, cgMLST‐relatedness alone cannot confirm the cross‐border spread, ancestral origin, or transmission direction. These questions require representative sampling, temporal and movement data, and higher‐resolution phylogenomic analyses.

A One Health perspective is relevant to brucellosis control in Southeast Asia [30]. The cgMLST dataset was limited to 24 profiles from Malaysia and Thailand. This geographic concentration may reflect uneven sequencing capacity, metadata availability, and data sharing rather than true regional differences in genetic diversity. A value‐chain modeling study from China identified inadequate quarantine and unregulated brokerage as important risk points for brucellosis introduction in cattle [31]. The findings of this review directly support wider genomic sampling, standardized metadata, and coordinated regional data exchange. Broader measures related to livestock movement, compensation, biosecurity, and postexposure management draw on the supporting literature and should be distinguished from the direct findings of this review.

Control measures should also consider the financial and social constraints of livestock‐dependent households because culling, movement restrictions, and animal replacement may discourage disease reporting. Environmental and market disruptions may further affect animal movement and access to health and veterinary services. More broadly, One Health control strategies may include coordinated human–animal surveillance, risk communication, accessible diagnostic and veterinary services, appropriate compensation, and cross‐border information exchange [32]. Operational measures may include targeted screening of high‐risk livestock populations, farm biosecurity, and appropriate postexposure management for occupationally exposed groups [33].

This review has several limitations. First, the search was restricted to English‐language publications and excluded local‐language reports, gray literature, and unpublished surveillance data. This restriction may have introduced language and publication bias, particularly in countries where surveillance findings are disseminated outside international journals. The absence of eligible studies should therefore be interpreted as a gap in accessible published evidence rather than proof of an absent surveillance activity or disease. Second, the evidence spans decades during which livestock systems, population mobility, markets, healthcare access, surveillance systems, and laboratory capacities have changed considerably. The study period was considered in the qualitative synthesis, but the sparse and uneven distribution of evidence across countries and time precluded formal temporal comparison; older estimates were therefore interpreted as historical evidence of occurrence rather than estimates of the current epidemiological situation. Third, many studies involved selected or high‐risk populations, and the MMAT assessment identified sampling and population representativeness as common methodological limitations. Differences in diagnostic approaches further limited the direct comparison of the reported crude seropositivity estimates. Fourth, data are sparse for several countries, for women and informal workers, and for pigs, buffaloes, wildlife, environmental sources, wildlife‐livestock interfaces, and mixed farming systems. The cgMLST analysis was limited to 24 profiles from two countries and was therefore insufficient for source attribution, transmission inference, or regional population structuring. Future studies should prioritize representative contemporary sampling, coordinated human–animal investigations, transparent diagnostic reporting, and broader inclusion of local‐language evidence and regional genomic data.

## 5. Conclusion

This review summarizes serological, microbiological, and molecular evidence on brucellosis in Southeast Asia, including seroprevalence in human and animal populations, bacterial isolation and species identification, and PCR‐ and cgMLST‐based characterization. The available evidence documents the circulation of *Brucella* in human and animal populations and reveals substantial gaps in published evidence, geographic coverage, species‐level identification, and genomic surveillance. Brucellosis remains an important zoonotic concern in Southeast Asia, although its regional epidemiology is still incompletely characterized. Improved human and animal surveillance, greater microbiological and genomic capacity, and sustained cross‐sectoral and cross‐country collaboration are needed to strengthen the risk assessment and support targeted prevention and control.

## Author Contributions

Qiuju Yang and Zhiguo Liu conceived the study and prepared the initial manuscript. Na Zhang and Linghui Yuan conducted the literature review and curated the epidemiological metadata. Xiaoan Cao and Zhenjun Li critically reviewed and revised the manuscript.

## Funding

This study was supported by the National Key Research and Development Program of China (Grant 2025YFD1800102).

## Disclosure

All authors reviewed and approved the final version. The funding body had no role in the design of the study, collection, analysis and interpretation of data, writing of the manuscript, or decision to submit the article for publication.

## Ethics Statement

This study was conducted in accordance with the principles of the Declaration of Helsinki. Ethics approval was not required because the review relied exclusively on published literature and publicly available, deidentified cgMLST records and involved no new human or animal sampling.

## Conflicts of Interest

The authors declare no conflicts of interest.

## Supporting Information

Additional supporting information can be found online in the Supporting Information section.

## Supporting information


**Supporting Information** Figure S1: Study selection flow diagram for the systematic review of brucellosis in Southeast Asia.

## Data Availability

All the epidemiological data summarized in this review are available in the cited publications and accompanying tables. The cgMLST profiles used for genomic contextualization are publicly available through PubMLST.
